# Estrogen Receptor α Signaling in Osteoblasts is Required for Mechanotransduction in Bone Fracture Healing

**DOI:** 10.3389/fbioe.2021.782355

**Published:** 2021-12-07

**Authors:** Lena Steppe, Benjamin Thilo Krüger, Miriam Eva Angelica Tschaffon, Verena Fischer, Jan Tuckermann, Anita Ignatius, Melanie Haffner-Luntzer

**Affiliations:** ^1^ Institute of Orthopedic Research and Biomechanics, University Medical Center Ulm, Ulm, Germany; ^2^ Institute of Comparative Molecular Endocrinology (CME), Ulm University, Ulm, Germany

**Keywords:** osteoblasts, estrogen receptor signaling, whole-body vibration, fracture healing, LMHFV, wnt signaling, prostaglandin signaling

## Abstract

Biomechanical stimulation by whole-body low-magnitude high-frequency vibration (LMHFV) has demonstrated to provoke anabolic effects on bone metabolism in both non-osteoporotic and osteoporotic animals and humans. However, preclinical studies reported that vibration improved fracture healing and bone formation in osteoporotic, ovariectomized (OVX) mice representing an estrogen-deficient hormonal status, but impaired bone regeneration in skeletally healthy non-OVX mice. These effects were abolished in general estrogen receptor α (ERα)-knockout (KO) mice. However, it remains to be elucidated which cell types in the fracture callus are targeted by LMHFV during bone healing. To answer this question, we generated osteoblast lineage-specific ERα-KO mice that were subjected to ovariectomy, femur osteotomy and subsequent vibration. We found that the ERα specifically on osteoblastic lineage cells facilitated the vibration-induced effects on fracture healing, because in osteoblast lineage-specific ERα-KO (ERα^fl/fl; Runx2Cre^) mice the negative effects in non-OVX mice were abolished, whereas the positive effects of vibration in OVX mice were reversed. To gain greater mechanistic insights, the influence of vibration on murine and human osteogenic cells was investigated *in vitro* by whole genome array analysis and qPCR. The results suggested that particularly canonical WNT and Cox2/PGE_2_ signaling is involved in the mechanotransduction of LMHFV under estrogen-deficient conditions. In conclusion, our study demonstrates a critical role of the osteoblast lineage-specific ERα in LMHFV-induced effects on fracture healing and provides further insights into the molecular mechanism behind these effects.

## Introduction

In bone repair, an optimal mechanical environment is required for the successful healing of the fractured bone (Claes et al., 1995; Haffner-Luntzer et al., 2015; Claes, 2017). Long-bone fracture healing critically depends on the stability and type of fracture fixation resulting in different degrees of interfragmentary movement, which either guide intramembranous or endochondral bone formation. A too rigid fixation with less interfragmentary movements results in lower strains around the fracture area and can even hinder successful healing (Perren, 1979; Claes et al., 1998). Clinical and preclinical studies suggest, that the endocrine status is also important because a disturbed bone formation during fracture healing is commonly observed in postmenopausal osteoporotic patients and OVX rodents (Kim et al., 2001; Nikolaou et al., 2009; Beil et al., 2010; He et al., 2011; Cheung et al., 2016). This can be explained by the importance of the osteoanabolic hormone estrogen for bone health (Cauley, 2015). Therefore, external mechanostimulation applied by whole-body low-magnitude high-frequency vibration (LMHFV) was suggested as a promising approach to improve compromised bone repair in osteoporotic subjects (Thompson et al., 2014; Edwards and Reilly, 2015). Several clinical trials investigating fracture healing and LMHFV are ongoing with no published results yet. Regarding the intact skeleton, clinical studies mainly reports anabolic effects of LMHFV on the osteoporotic skeleton (Rubin et al., 2004), whereas one study demonstrates no effect on bone mineral density (Leung et al., 2014). Preclinical studies demonstrate that LMHFV application provokes positive effects on bone healing in sheep (Li et al., 2018) and in ovariectomy-induced estrogen-deficient rodents (Stuermer et al., 2010; Komrakova et al., 2013; Chung et al., 2014; Wehrle et al., 2015; Wang et al., 2017; Steppe et al., 2020). By contrast, no or negative effects on bone repair were observed in non-OVX rodents (Shi et al., 2010; Stuermer et al., 2010; Chung et al., 2014; Wehrle et al., 2014, 2015; Wang et al., 2017; Steppe et al., 2020).

Therefore, estrogen seems to play an important role in regulating the mechanotransduction of LMHFV during fracture healing. The effect of estrogen on bone is mainly mediated via its interaction with two estrogen receptors (ERs), ERα and ERβ. Both are expressed by chondrocytes and osteogenic cells (Manolagas et al., 1995; Almeida et al., 2017), but regulate the expression of different target genes. In addition to the classic ER pathway involving ligand-binding and the subsequent interaction with transcription factors or estrogen-responsive elements or, also ligand-independent signaling can be exerted by ERα and ERβ in the absence of estrogen. Several studies report that ERα expression is decreased in the fracture callus of OVX mice compared to non-OVX animals (He et al., 2011), whereas mechanical stimulation enhanced ERα expression in the fracture callus particularly in OVX rodents (Wehrle et al., 2015; Chow et al., 2016; Haffner-Luntzer et al., 2018a). Furthermore, other reports showed that ERα is required for mediating the anabolic effects of mechanical strain (Lee et al., 2003; Jessop et al., 2004; Windahl et al., 2013; Chow et al., 2016), indicating that ERα is involved in mechanotransduction in bone. By using a global ERα-knockout (ERα-KO) mouse model, we previously demonstrated that in the absence of ERα, both the improved fracture healing and bone formation upon vibration in OVX animals and the impaired bone regeneration in non-OVX mice were not observed anymore (Haffner-Luntzer et al., 2018a). ERβ-KO did not display any effect, indicating only a minor role of this receptor-however, by using global KO mice we were unable to conclude about systemic versus local effects of ERα during mechanostimulation in fracture healing. Notably, we found that the number and the contact area between osteoblasts and bone (osteoblast surface) in the fracture callus of wildtype mice were decreased in non-OVX but increased in OVX animals by vibration, whereas these parameters were unaltered in ERα-KO OVX mice upon LMHFV treatment (Haffner-Luntzer et al., 2018a). Since osteoblasts are involved in bone regeneration (Dirckx et al., 2013; Bahney et al., 2019) and as ERα is highly expressed by osteoblasts (Haffner-Luntzer et al., 2018a), we hypothesized that osteoblasts are direct target cells of LMHFV via ERα signaling. To investigate this hypothesis, we generated mice with an osteoblast lineage-specific ERα-KO (ERα^fl/fl; Runx2Cre^), which underwent ovariectomy followed by a femur osteotomy and LMHFV treatment.

## Materials and Methods

### Animal Care and Animal Models

All experiments were performed according to the German Guidelines of Animal Research on the Protection of Animals as well as the ARRIVE guidelines and were approved by the local ethical committee (No. 1455, Regierungspräsidium Tübingen, Germany). Osteoblast lineage-specific ERα-KO mice (Tg (Runx2-cre)1Jtuc x Esr1^tm1.2Mma^) were generated by crossing Runx2-Cre with ERα^fl/fl^ mice on a C57BL/6 background, both provided by Prof. J. Tuckermann (Ulm University). Cre^−^ littermates (ERα^fl/fl^) were used as control. Cre^+^ mice (ERα^fl/fl; Runx2Cre^) were shown to lack the ERα in cells of the osteogenic lineage (Supplementary Figure S1) including osteoblasts, osteocytes and hypertrophic chondrocytes. All animals were housed in groups of up to five mice per cage with a 12 h light, 12 h dark rhythm, and received water *ad libitum* as well as a standard mouse feed (Ssniff R/M-H, V1535-300; Ssniff, Soest, Germany) until the day of ovariectomy/sham-ovariectomy. Subsequently, the food was switched to a phytoestrogen-free diet (Ssniff). Mouse genotyping was conducted by lysed ear punch PCR using the primers: 5′-CCA​GGA​AGA​CTG​CCA​GAA​GG-3′, 5′-TGGCTTGCAGGT ACAGGAG-3′ and 5′-GGA​GCT​GCC​GAG​TCA​ATA​AC-3′ to detect the Cre transgene, whereas the ERα loxP sites were detected using the primers: 5′- TAGGCTTTGTCTCGCTTT CC-3′, 5′- CCCTGG CAAGATAAGACAGC-3′ and 5′-AGG​AGA​ATG​AGG​TGG​CAC​AG-3′.

### Surgical Procedures

When aged 12 weeks, female mice (*n* = 24 per genotype) were randomly assigned (Table 1) to bilateral ovariectomy (*n* = 12 per genotype) or sham-operated (non-OVX, *n* = 12 per genotype) as described previously (Haffner-Luntzer et al., 2017). 4 weeks after OVX or non-OVX surgery, all mice underwent standardized unilateral femur osteotomy as described previously (Röntgen et al., 2010; Wehrle et al., 2015; Haffner-Luntzer et al., 2017). Briefly, the osteotomy was created at the right femur diaphysis using a 0.4 mm Gigli wire saw (RISystem, Davos, Switzerland) and stabilized by a semi-rigid external fixator (RISystem). Half of the OVX (*n* = 6 per genotype) and non-OVX mice received LMHFV. Three weeks after osteotomy surgery (day 21), all mice were sacrificed using an isoflurane overdose and cardiac blood withdrawal.

**TABLE 1:** Experimental treatment groups per each genotype (ERα^fl/fl^, ERα^fl/fl; Runx2Cre^).

**Group**	**OVX**	**Osteotomy**	**LMHFV**
*Non-OVX*	−	+	−
*Sham Vibration*			
*Non-OVX Vibration*	−	+	+
*OVX*	+	+	−
*Sham Vibration*			
*OVX*	+	+	+
*Vibration*			

### LMHFV

**FIGURE 1:**
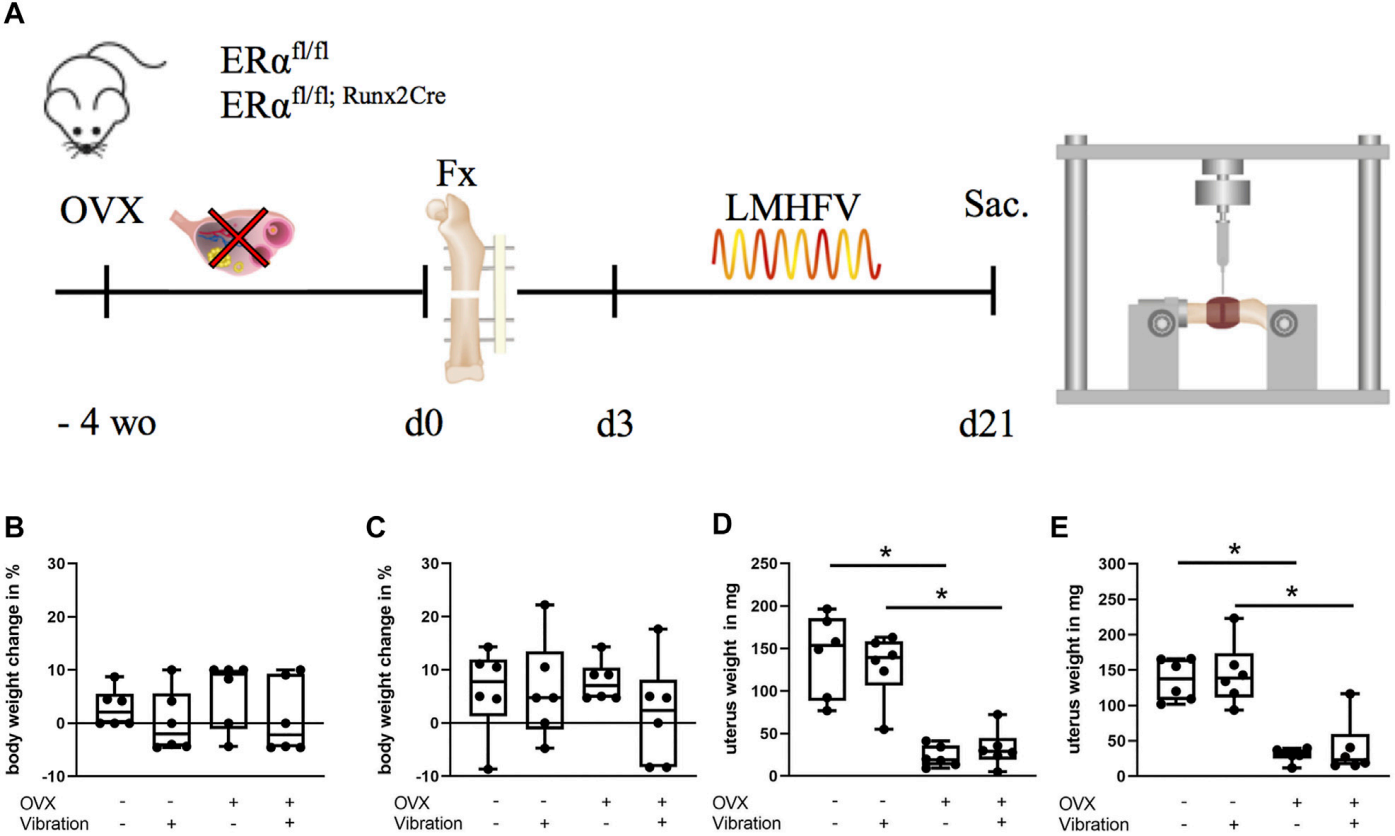
Schematic illustration of the experimental timeline and physiological parameters of ERα^fl/fl^ and ERα^fl/fl; Runx2Cre^ mice. **(A)** When aged 12 weeks, mice were ovariectomized (OVX) or sham-operated followed by fracture surgery (Fx) 4 weeks later. Vibration (LMHFV) or sham-vibration treatment started on the third postoperative day for 20 min/day and 5 days/week. On day 21 after fracture, the mice were sacrificed (Sac.) and their bones were analyzed. Body weight of ERα^fl/fl^ **(B)** and ERα^fl/fl; Runx2Cre^ **(C)** mice as well as their respective uterus weight [**(D):** ERα^fl/fl^, **(E):** ERα^fl/fl; Runx2Cre^] was assessed. Data are shown as box-and-whisker plots (with median and interquartile range) from max. to min., showing all data points. *indicates significant effects with *p* ≤ 0.05.

The LMHFV regimen was chosen due to our previous studies, showing that 45 Hz significantly improved fracture healing in OVX mice (Wehrle et al., 2015; Haffner-Luntzer et al., 2018a). Starting on the third day after osteotomy surgery, mice were placed on custom-made vibration platforms for 20 min per day for 5 days per week and received vertical whole-body vibration with 0.3 g sinusoidal peak-to-peak acceleration and 45 Hz frequency, as described previously (Wehrle et al., 2015) (Figure 1A). The amplitude and frequency were continuously recorded using integrated accelerometers at the platform (Sensor KS95B.100, measurement amplifier Innobeamer L2, Software Vibromatrix; IDS Innomic GmbH, Salzwedel, Germany). The control mice were sham-vibrated on the same platforms without activation of the vibration generator. All mice were allowed to move freely on the platforms during the vibration or sham-vibration treatment and afterwards returned to their home cages.

### Biomechanical Testing and μCT Analysis

To evaluate the mechanical properties of intact and osteotomized femurs explanted on day 21, a non-destructive three-point bending test was performed as described previously (Röntgen et al., 2010; Wehrle et al., 2015). Briefly, following removal of the fixator, an axial load with a maximum of 2 N was applied on top of the callus side in a cranio-lateral position using a material-testing machine (1454, Zwick GmbH & Co KG, Ulm, Germany). The bending stiffness was calculated from the slope of the load-deflection curve as described previously (Röntgen et al., 2010). If the bones were too fragile for reaching a preload of 0.05 N, the three-point bending test was not performed and a flexural rigidity value of 0 was given. Following biomechanical testing, femora were fixed in 4% phosphate-buffered formaldehyde solution and scanned using a μCT scanning device (Skyscan 1172 version 1.5; Skyscan, Kontich, Belgium) at a resolution of 8 μm using a peak voltage of 50 kV and 200 μA. Analyses and calibration steps were performed according to the guidelines of the American Society for Bone and Mineral Research (Bouxsein et al., 2010). The volume of interest was defined as the entire callus between the fractured cortices. Within each scan, two phantoms with a defined density of hydroxyapatite (250 and 750 mg hydroxyapatite/cm^3^) were included to determine the bone mineral density. To distinguish between mineralized and nonmineralized tissue, a threshold for cortical bone set at 641.9 mg hydroxyapatite/cm^3^ was used (Morgan et al., 2009). The trabecular bone of the intact left femora was assessed 200 μm proximal of the metaphyseal growth plate over a length of 280 μm in the distal femur, excluding the cortex. For assessing the trabecular bone parameters, a threshold set at 394.8 mg hydroxyapatite/cm^3^ was used (Morgan et al., 2009). Analyses were performed by means of Skyscan software (NRecon version 1.7.1.0, DataViewer version 1.5.1.2, and CTAn version 1.17.2.2). Bony bridging score was determined in two perpendicular planes with one bridged cortex counting for one scoring point. A bony bridging score of 3 or 4 represented successful healing.

### Histomorphometry of the Fracture Callus and Immunohistochemical Staining

Following μCT analysis, bone specimens were subjected to decalcified histology as described previously (Haffner-Luntzer et al., 2014). Sections of 7 μm were stained with Safranin O for histomorphometric tissue quantification. The amounts of bone, cartilage and fibrous tissue in the fracture gap were determined using an image analysis software (Leica DMI6000 B; Software MMAF Version 1.4.0 MetaMorph^®^; Leica, Heerbrugg, Switzerland). The number and surface of osteoclasts (NOc/BPm, OcS/BS) were quantitatively assessed using tartrate-resistant alkaline phosphatase (TRAP) staining. Osteoclasts were defined as TRAP-positive cells with two or more nuclei, directly located on the bone surface with a visible resorption lacuna between the bone matrix and the cell. The number and surface of osteoblasts were determined using Safranin-O staining. Osteoblasts were defined as cubic-shaped cells with visible cytoplasm, located directly on the bone surface. Bone cells and surface were evaluated in a rectangular area (650 × 450 μm) using the Osteomeasure system (Osteometrics, Decatur, United States). Images were obtained using a Leica microscope (DMI 6000B). All analyses were performed according to the American Society for Bone and Mineral Research guidelines for bone histomorphometry (Dempster et al., 2013).

For immunohistochemical staining, paraffin-embedded sections of 4 µm were deparaffinized, enzymatically demasked by trypsin, blocked with 3% peroxidase followed by a serum block with 5% goat serum in TBS-T for 2 h. Staining for ERα was performed at 4°C overnight using the following primary antibody: rabbit anti-mouse ERα (1:75, # PA5-16440, Invitrogen). Rabbit IgG was included as isotype control to confirm specific staining. Next, sections were washed with TBS and incubated with a goat anti-rabbit secondary antibody (1:100, #B2770, Life Technologies) for an hour at RT. After another washing step, horseradish peroxidase (HRP)-conjugated streptavidin (#PK-6100, VECTASTAIN^®^ Elite ABC-HRP Kit, Peroxidase, Vector Laboratories) was applied according to the manufacturer`s guidelines. NovaRED (#SK-4800, Vector^®^ NovaRED^®^ Substrate Kit, Peroxidase (HRP), Vector laboratories) was used as chromogen and the sections were counterstained with hematoxylin (1:5; #2C-306, Waldeck, Münster, Germany) and rinsed. This staining protocol was previously established by using biological negative controls (bone sections from ERα-general KO mice). In ERα-stained sections, osteoblasts were identified by their cubic shape and direct contact to the bone trabeculae, whereas osteocytes were defined as ellipsoidal shaped cells embedded in the mineralized bone matrix. Hypertrophic chondrocytes were identified as spherical or polygonal shaped cells within a population of other chondrocytes.

### Cell Culture Experiments and Transcriptome Analysis

Murine MC3T3-E1 cells were purchased from the American Type Culture Collection (ATCC) and seeded at a density of 4,000 cells per cm^2^. On day 3 after seeding, cells were differentiated by adding 50 mg/ml ascorbic acid and 10 mM β-glycerophosphate to the phenol-red free culture medium containing 10% charcoal-stripped (estrogen-free) fetal calf serum (FCS), 1% L-glutamine and 1% penicillin/streptomycin (all ThermoFisher Scientific). Half of the cultures were subjected to LMHFV on a custom-made vibration platform at 0.3 g peak-to-peak acceleration/45 Hz for 20 min/day for 5 days. The other half of the cultures (sham-vibrated) were placed on the same platform without turning on the vibration device. Total RNA was isolated as described previously (Haffner-Luntzer et al., 2018b) and microarray-based gene expression analysis with mouse Gene 1.0 ST GeneChip^®^ arrays was performed as published previously (Mödinger et al., 2018). Differentially expressed probesets were determined by *t* test and considered statistically significant when *p* < 0.05 and fold change ≤1.5, as described previously (Zoller et al., 2017). To identify the involved molecular pathways, the GoMINER tool (Zeeberg et al., 2003) was used, whereas functional protein interaction networks were identified using the STRING 10 program (http://string-db.org/). Complete microarray data are available in the Supplementary Material. For the Cox2 antagonist experiment, celecoxib was obtained from Sigma (SML3031) and prepared using dimethyl sulfoxide (stock: 2 mg/ml). AH23848 (EP4 antagonist) was purchased from Cayman Chemical (19023) and dissolved in dimethyl sulfoxide (stock: 5 mg/ml). Both antagonists were used at a concentration of 1 × 10^−6^ M.

Human bone marrow-derived mesenchymal stem cells (hMSCs) were obtained by Lonza (PT-2501) and seeded in 24-well plates (10,000 cells/well) 2 days prior to the differentiation experiments. The medium was changed to differentiation medium (phenol-red free α-MEM, 10% FCS + 1% penicillin/streptomycin + 1% L-glutamine, 50 mg/ml ascorbic acid, 10 mM β-glycerophosphate) at day 0 and the cells were allowed to differentiate for 10 days. At day 10, the medium was switched to estrogen-free medium and vibration was performed 4 h after the medium-switch for 20 min at 45 Hz and 0.3 g. Thirty minutes after the end of the vibration treatment, the experiment was stopped and RNA was isolated (RNeasy Mini kit, Qiagen) for analyzing gene expression by qPCR and human Gene 1.0 ST GeneChip^®^ arrays.

### qPCR

The SensiFAST SYBR Hi-ROX One-Step Kit (Bioline, Mempis, United States) was used according to the manufacturer’s guidelines to perform quantitative PCR. *B2M* (murine F: 5′-ATA​CGC​CTG​CAG​AGT​TAA​GCA-3′, murine R: 5′-TCA​CAT​GTC​TCG​ATC​CCA​GT-3′, human F: 5′-CTC​ACG​TCA​TCC​AGC​AGA​GA-3′, human R: 5′-GGA​TGG​ATG​AAA​CCC​AGA​CA-3′) was used as the housekeeping gene. Relative gene expression of *Lef1* (murine F: 5′-TCA CCT ACA GCG ACG AG-3′, murine R: 5′-TGA​CAT​CTG​ACG​GGA​TGT​GT-3′, human F: 5′ GAG​ATT​TCT​CTG​TAT​GGC​ACC-3′, human R: 5′-CTG​CAA​TGA​GAC​ACT​TTC​TC-3′), *Ptgs2* (murine F: 5′-AGG​GGT​GTC​CCT​TCA​CTT​CT-3′, murine R: 5′-CAT​TGA​TGG​TGG​CTG​TTT​TG-3′, human F: 5′-TAA​GGG​GAG​AGG​AGG​GAA​AA-3′, human R: 5′-CTG​CTG​AGG​AGT​TCC​TGG​AC-3′) and *Mdk* (human F: 5′-TGC​CCT​GCA​ACT​GGA​AGA​A-3′, human R: 5′-GCCTGTGCCCCC ATCAC-3′) was calculated using the delta-delta CT method.

### Statistics

Group size was *n* = 6 per group and per genotype (ERα^fl/fl^, ERα^fl/fl; Runx2Cre^). In one animal, the histological sections were not evaluable, therefore analyzing this group with *n* = 5 (Figures 3E–H, second boxplot). Data were tested for normal distribution using the Shapiro-Wilk test, and data sets were normally distributed. Statistical testing between two groups was done using two-tailed Student’s t-test, whereas comparisons between more than two groups were performed by one-way ANOVA with post hoc Tukey’s test for adjusting the *p*-value for multiple comparisons using GraphPad Prism 8.4.3 (GraphPad Software, La Jolla, CA). The level of significance was set at *p* ≤ 0.05. Results are presented as box-and-whisker plots (with median and interquartile range) from max. to min., showing all data points.

## Results

### Body Weight and Uterus Weight Changes

All mice received the selected treatments including ovariectomy/sham-operation, fracture and LMHFV/sham-vibration as illustrated in Figure 1A. The body weight of the animals was not significantly influenced by the treatments or the genotype during the entire experimental period (Figures 1B,C). To control for the success of OVX in ERα^fl/fl^ and ERα^fl/fl; Runx2Cre^ mice, uterus weight at day 21 after osteotomy was assessed. In both mouse strains, OVX resulted in a significantly lower uterus weight (Figures 1D,E). Additionally, we performed µCT analysis of the intact left femora to determine if our mice developed an osteoporotic bone phenotype after OVX treatment. We found that non-vibrated and vibrated OVX groups from both genotypes displayed lower bone volume fraction and trabecular number, either significantly or by strong trend (Supplementary Figure S2).

### Fracture Healing in Control (ERα^fl/fl^) Mice

**FIGURE 2:**
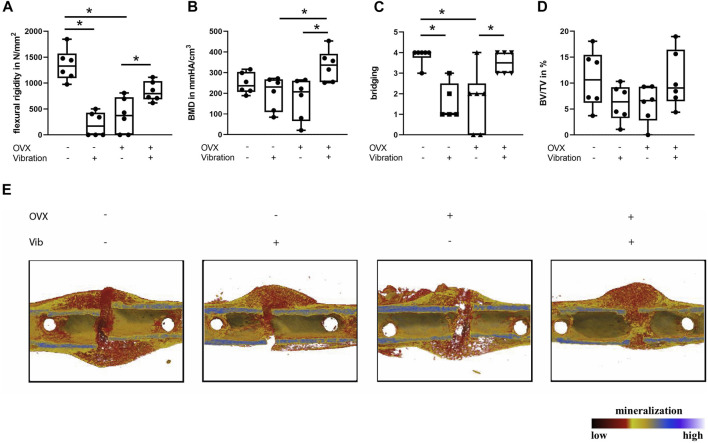
Influence of LMHFV on fracture healing in ERα^fl/fl^ mice on day 21. Biomechanical testing and μCT analysis. **(A)** Bending stiffness of the fracture callus. **(B)** Bone mineral density of the fracture gap. **(C)** Bony bridging of the fracture gap evaluated in two perpendicular planes. **(D)** Relative bone volume (BV/TV) in the fracture gap. **(E)** Representative μCT images from the fractured femurs. Red indicates weakly mineralized bone, whereas yellow and light blue indicate highly mineralized bone. Data are shown as box-and-whisker plots (with median and interquartile range) from max. to min., showing all data points. *indicates significant effects with *p* ≤ 0.05.

**FIGURE 3:**
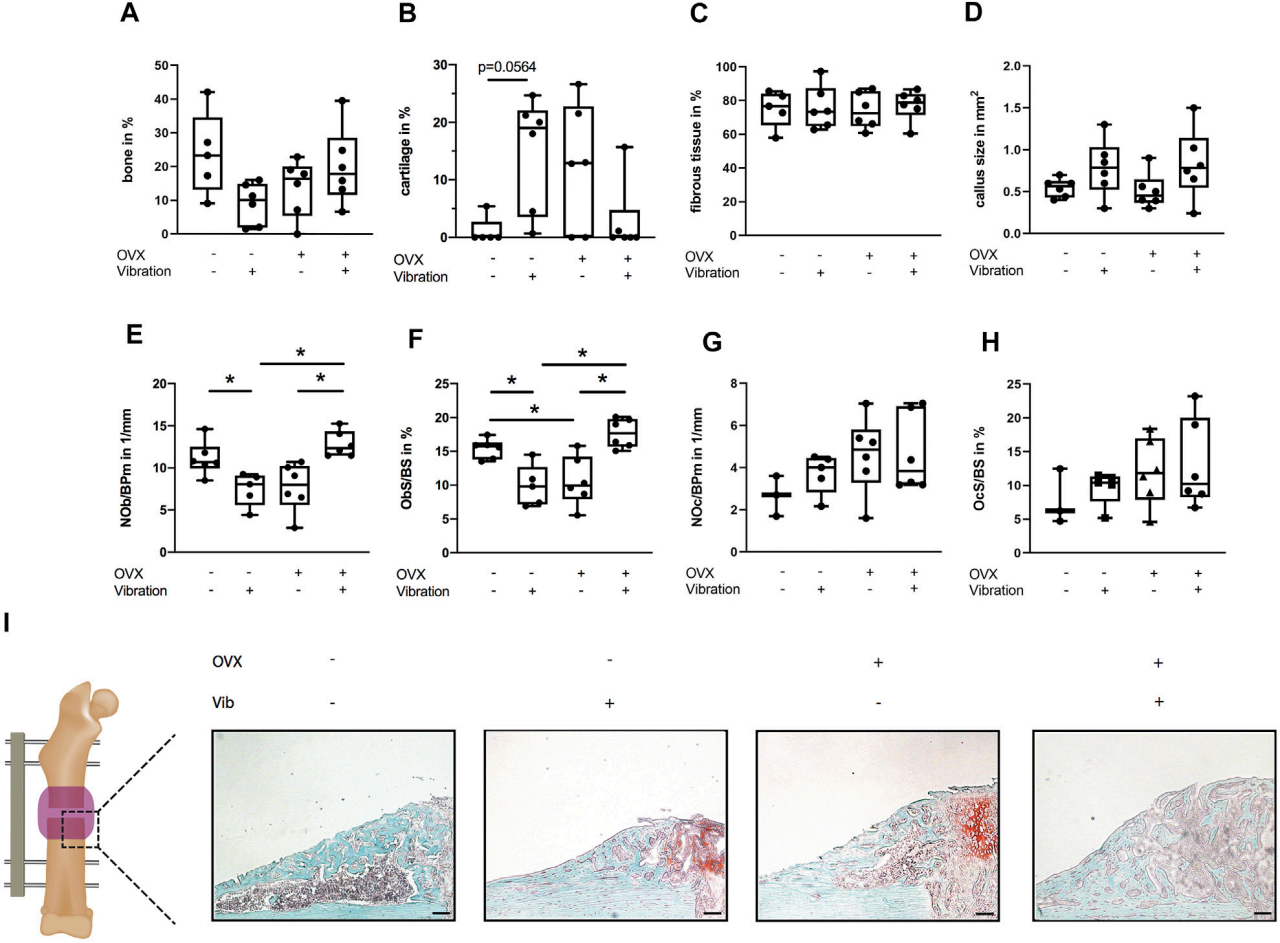
Histomorphometrical analysis of the fracture gap in ERα^fl/fl^ mice on day 21. Paraffin sections of fractured femurs were stained with Safranin O and histomorphometrical analysis was performed to determine **(A)** relative bone area, **(B)** relative cartilage area, **(C)** relative fibrous tissue area and **(D)** callus size. **(E)** Number of −6+s per bone perimeter (NOb/BPm) and **(F)** osteoblast surface per bone surface (ObS/BS) in the fracture gap. **(G)** Number of osteoclasts per bone perimeter (NOc/BPm) and **(H)** osteoclast surface per bone surface (OcS/BS). **(I)** Representative images from the fracture callus at day 21 after surgery stained with Safranin O. Scale bar = 100 μm. Data are shown as box-and-whisker plots (with median and interquartile range) from max. to min., showing all data points. *indicates significant effects with *p* ≤ 0.05.

In non-OVX mice that received LMHFV treatment, a significant reduction of flexural rigidity (*p* < 0.0001) and bony bridging (*p* = 0.0047) of the fracture gap was displayed, whereas the bone mineral density and the relative bone volume ratio did not differ significantly (Figures 2A–D). Furthermore, histomorphometrical analysis revealed a by trend reduced relative bone area (*p* = 0.0923), an increased cartilage area (*p* = 0.0564) as well as a significantly reduced osteoblast count (*p* = 0.0539) and a significantly lower osteoblast activity (*p* = 0.0159) in vibrated non-OVX ERα^fl/fl^ mice (Figures 3A–F). All these data indicated impaired fracture healing at day 21 in non-OVX mice subjected to vibration treatment.

Bone regeneration was also compromised in OVX mice compared to non-OVX mice, as indicated by an inferior flexural rigidity (*p* < 0.0001), significantly reduced bony bridging (*p* = 0.0041) and by trend increased amount of cartilage (*p* = 0.1469) in the fracture callus (Figures 2, 3). Similarly, osteoblast number (*p* = 0.0576) and surface (*p* = 0.0296) were also diminished in OVX mice compared to non-OVX mice (Figures 3E,F).

Bone healing was improved by LMHFV in OVX mice in comparison to OVX animals as indicated by significantly enhanced flexural rigidity (*p* = 0.0374), bone mineral density (*p* = 0.0108) and bony bridging (*p* = 0.0155) of the fracture gap and a by trend reduced cartilage area (*p* = 0.2248) (Figures 2, 3). Furthermore, LMHFV resulted in a significantly increased number (*p* = 0.0031) and activity of osteoblasts (*p* = 0.0012) in OVX ERα^fl/fl^ mice compared to OVX animals without vibration (Figures 3E,F). The callus size as well as the number and surface of osteoclasts did not differ significantly between all the ERα^fl/fl^ groups (Figures 3D,G,H). Overall, these data demonstrated that OVX and LMHFV both disturb bone regeneration in control ERα^fl/fl^ mice, whereas in combination both treatments resulted in an improved fracture healing.

### Fracture Healing in Osteoblast-Specific ERα-KO (ERα^fl/fl; Runx2Cre^) Mice

Although mice with a specific deletion of the ERα in the osteoblast lineage (ERα^fl/fl; Runx2Cre^) were previously shown to have a preexisting bone phenotype with decreased trabecular bone mass in the spine and tibiae (Seitz et al., 2012), we found that this does not influence fracture healing (*p* = 0.439) when directly comparing non-OVX non-vibrated animals of both genotypes (Supplementary Figure S3).

**FIGURE 4:**
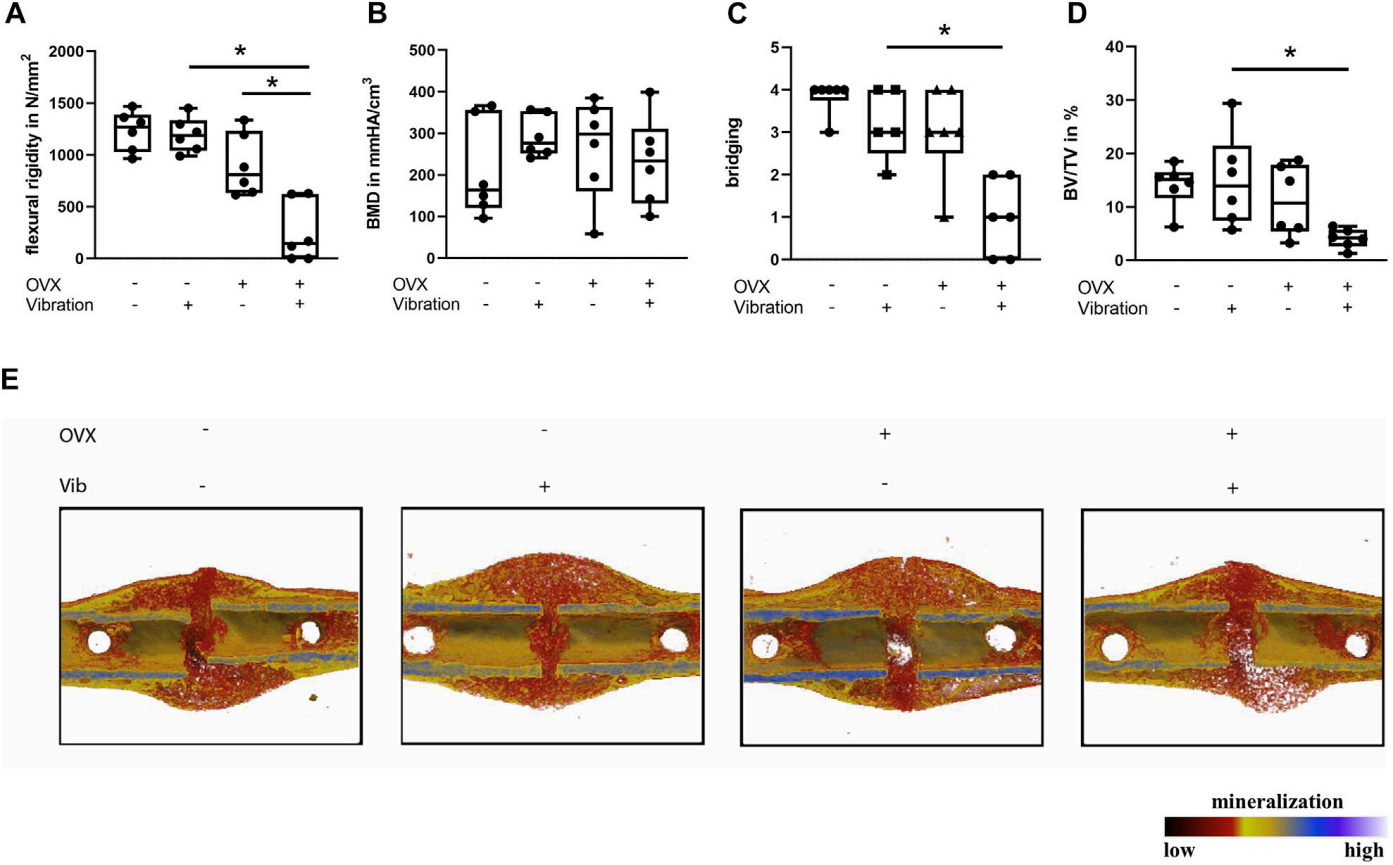
Influence of LMHFV on fracture healing in ERα^fl/fl; Runx2Cre^ mice on day 21. Biomechanical testing and μCT analysis. **(A)** Bending stiffness of the fracture callus. **(B)** Bone mineral density of the fracture gap. **(C)** Bony bridging of the fracture gap evaluated in two perpendicular planes. **(D)** Relative bone volume (BV/TV) in the fracture gap. **(E)** Representative μCT images from the fractured femurs. Red indicates weakly mineralized bone whereas yellow and light blue indicate highly mineralized bone. Data are shown as box-and-whisker plots (with median and interquartile range) from max. to min., showing all data points. *indicates significant effects with *p* ≤ 0.05.

**FIGURE 5:**
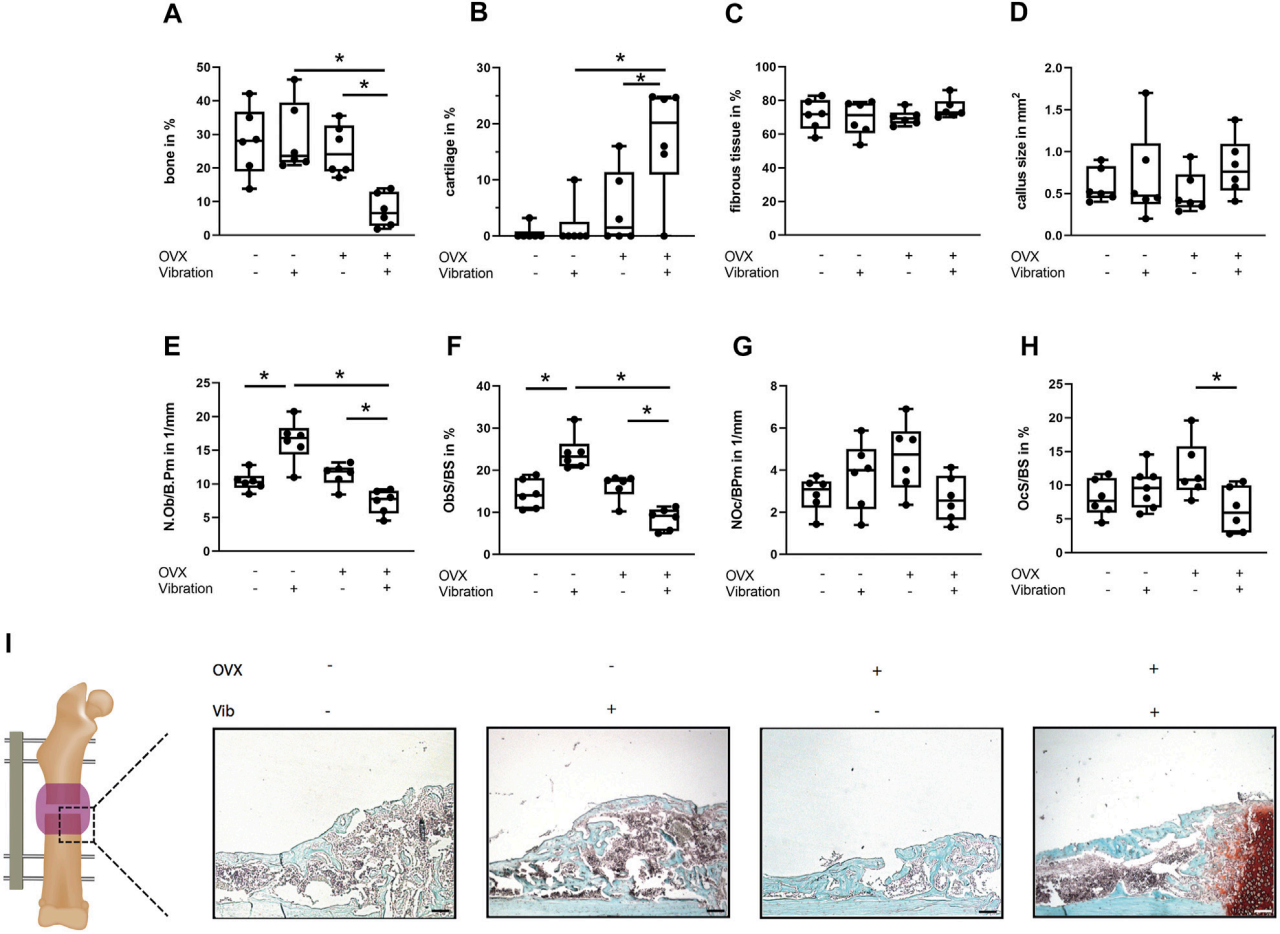
Histomorphometrical analysis of the fracture gap in ERα^fl/fl; Runx2Cre^ mice on day 21. Paraffin sections of fractured femurs were stained with Safranin O and histomorphometrical analysis was performed to determine **(A)** relative bone area, **(B)** cartilage area, **(C)** relative fibrous tissue area and **(D)** callus size. **(E)** Number of osteoblasts per bone perimeter (NOb/BPm) and **(F)** osteoblast surface per bone surface (ObS/BS) in the fracture gap. **(G)** Number of osteoclasts per bone perimeter (NOc/BPm) and **(H)** osteoclast surface per bone surface (OcS/BS). **(I)** Representative images from the fracture callus at day 21 after surgery stained with Safranin O. Scale bar = 100 μm. Data are shown as box-and-whisker plots (with median and interquartile range) from max. to min., showing all data points. *indicates significant effects with *p* ≤ 0.05.

Furthermore, LMHFV did not exert negative effects on fracture healing in non-OVX ERα^fl/fl; Runx2Cre^ mice, because there were no significant differences in flexural rigidity, bone content or bridging of the fracture gap in comparison to ERα^fl/fl; Runx2Cre^ mice that did not receive LMHFV (Figure 4). In addition, histomorphometrical parameters were unaltered by LMHFV treatment in non-OVX mice except the osteoblast number (*p* = 0.0005) and surface (*p* = 0.0004) that were significantly increased in vibrated non-OVX mice lacking the ERα in osteoblasts (Figure 5).

ERα^fl/fl; Runx2Cre^ OVX mice without vibration were protected from impaired fracture healing due to OVX, because a similar flexural rigidity, bony bridging and bone formation in the fracture callus was observed compared to non-OVX ERα^fl/fl; Runx2Cre^ mice (Figure 4). Furthermore, histomorphometrical analysis did not reveal any significant differences in callus composition or cellular parameters compared to non-OVX ERα^fl/fl; Runx2Cre^ mice (Figure 5).

Interestingly, reversed effects of vibration on bone formation during fracture healing in OVX ERα^fl/fl; Runx2Cre^ mice were observed, which resulted in a considerably reduced flexural rigidity (*p* = 0.001), by trend diminished bone formation (*p* = 0.1975) and significantly reduced bony bridging (*p* = 0.0032) compared to OVX ERα^fl/fl; Runx2Cre^ mice (Figures 4A,C,D) in contrast to the improved fracture healing seen in vibrated OVX control ERα^fl/fl^ mice. Histomorphometrical analysis further revealed a significantly increased cartilage area (*p* = 0.0114) in the fracture callus of OVX ERα^fl/fl; Runx2Cre^ animals subjected to vibration treatment (Figure 5B). In addition, the osteoblast surface (*p* = 0.0039) and number (*p* = 0.0188) were significantly decreased in vibrated OVX ERα^fl/fl; Runx2Cre^ mice (Figures 5E,F), whereas osteoclast activity was significantly reduced (*p* = 0.0315) (Figure 5H).

### Molecular Mechanisms

**FIGURE 6:**
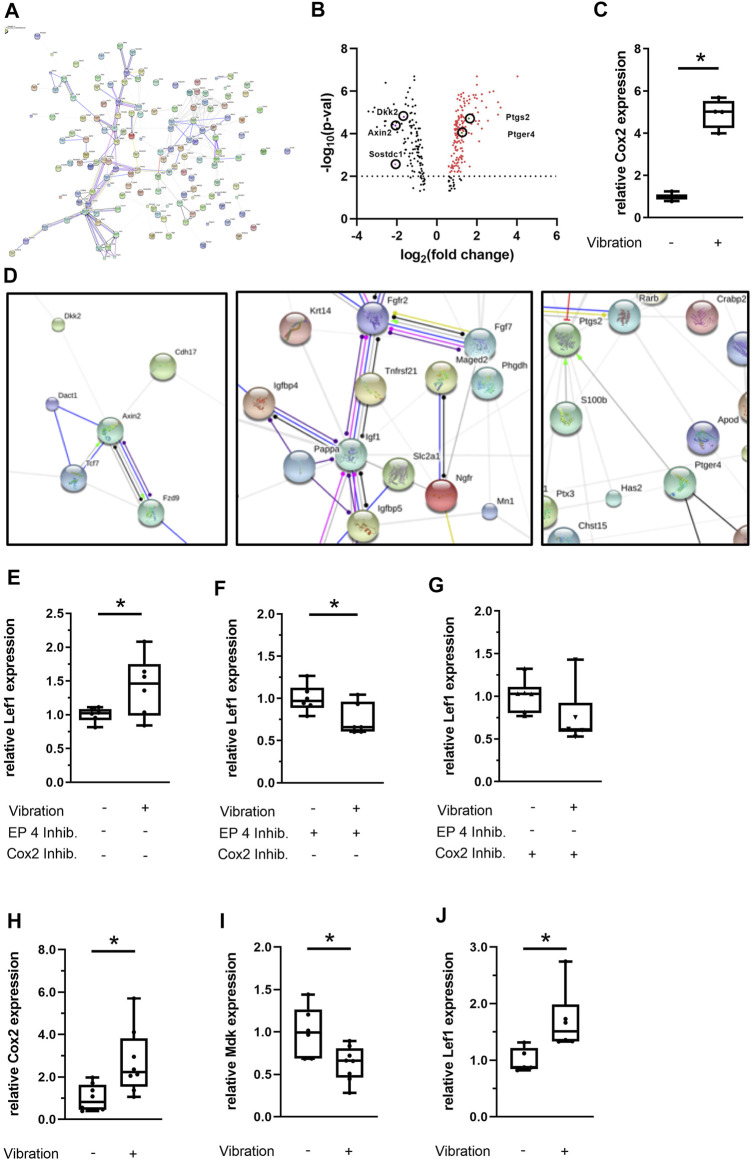
Transcriptome analysis of MC3T3-E1 cells and gene expression analysis of hMSCs upon vibration treatment under estrogen-free conditions. **(A)** Nondetailed STRING protein-association network (action) derived from the MC3T3-E1 microarray data. The image in high quality can be found in the supplemental material (Supplementary Figure S4). **(B)** Vulcano plot of differentially regulated genes and **(C)** qPCR validation of Cox2 expression in MC3T3-E1 cells. **(D)** Detailed STRING network (MC3T3-E1 microarray). **(E–G)** Effect of LMHFV in MC3T3-E1 cells pretreated with the EP4 antagonist AH23848 (1 × 10^−6^ M) or with the Cox2 antagonist celecoxib (1 × 10^−6^ M). **(H–J)** Effect of LMHFV in hMSCs. Data are shown as box-and-whisker plots (with median and interquartile range) from max. to min., showing all data points. *N* = 4–6 per group (vibrated and sham-vibrated), *indicates significant effects with *p* ≤ 0.05.

We performed whole genome microarray-based gene expression analysis of vibrated and sham-vibrated samples and found that 304 genes were differentially regulated upon LMHFV treatment (Figure 6). Among the 184 upregulated genes, the mechanosensitive enzyme Cox2, encoded by the gene *Ptgs2*, appeared in our array (Table 2), as well as bone remodeling and extracellular matrix-associated genes (Table 3). Downregulated genes were found to be involved in Wnt/β-catenin signaling (Table 4), with most of the genes encoding for proteins that act as Wnt inhibitors (*Axin2, Sostdc1, Dkk2*) except the gene encoding for the Wnt receptor *Fzd9*. STRING analysis was performed to investigate the interactions of the respective pathways (Figures 6A,D). As expected, the Wnt pathway genes were clustering as well as the genes for extracellular matrix and Cox2/PGE_2_ signaling (Figure 6D). Confirming the microarray findings, gene expression of Cox2 was validated by qPCR (Figure 6C). Consequently, we were interested whether the Cox2/PGE_2_ pathway might interact with canonical Wnt signaling. To investigate whether the signaling via the Cox2/EP4 axis might affect downstream signaling of the Wnt pathway, we performed further *in vitro* experiments with MC3T3-E1 cells pretreated with either the EP4 inhibitor AH23848 or the Cox2 inhibitor celecoxib. We demonstrated that the prostaglandin pathway via EP4 positively modulates the Wnt target gene *Lef1* in response to LMHFV, because the EP4 and Cox2 inhibitors abolished the LMHFV-induced *Lef1* upregulation (Figures 6E–G). Because we aimed to study the effects of LMHFV also in human cells, hMSCs were subjected to vibration and gene expression was assessed. We demonstrated that Cox2 in hMSCs was also upregulated by vibration (Figure 6H). Furthermore, the Wnt inhibitor *Mdk* was downregulated upon LMHFV, whereas the Wnt transcription factor *Lef1* was significantly upregulated (Figures 6I,J). In addition, microarray-based gene analysis of vibrated vs. non-vibrated hMSCs revealed that a further 56 genes were differentially regulated upon vibration. After excluding non-coding transcripts, 26 probesets remained for analysis. Among them, there were five micro RNAs (miRNAs) that are known to be involved in regulating bone metabolism, whereof three were upregulated and two downregulated (Table 5).

**TABLE 2:** Selected differentially regulated mechanosensitive genes upon vibration treatment in MC3T3-E1 cells under estrogen-free conditions.

**Gene**	**Description**	**Pathways**	**GenBank accession number**	***p*-value**	**Fold change**
*Ptgs2*	Prostaglandin-endoperoxide synthase 2	Eicosanoid metabolism, arachidonic acid metabolism, VEGF signaling pathway	NM_011198	1.92 × 10^−5^	3.03 ↑
*Ptger4*	Prostaglandin E receptor 4 (subtype EP4)	Eicosanoid metabolism	NM_001136079	9.12 × 10^−5^	2.38 ↑

**TABLE 3:** Selected differentially regulated bone remodeling and ECM-associated genes upon vibration treatment in MC3T3-E1 cells under estrogen-free conditions.

**Gene**	**Description**	**Pathways**	**GenBank accession number**	***p*-value**	**Fold change**
*Igf1*	Insulin-like growth factor 1	IGF-1 signaling pathway, focal adhesion	NM_001111274	1.625 × 10^−4^	2.04 ↑
*Spp1*	Secreted phosphoprotein 1	Regulators of bone mineralization, ECM-receptor interaction, focal adhesion, Toll-like receptor signaling pathway	NM_001204201	4.94 × 10^−5^	2.13 ↑
*Fosl1*	Fos-like antigen 1	Osteoclast differentiation, Wnt signaling pathway	NM_010235	1.008 × 10^−4^	2.33 ↑
*Ctsk*	Cathepsin K	Osteoclast differentiation, Toll-like receptor signaling pathway	NM_007802	1.436 × 10^−3^	0.60 ↓
*Fgf7*	Fibroblast growth factor 7	MAPK signaling pathway, regulation of actin cytoskeleton	NM_008008	5.29 × 10^−5^	2.56 ↑
*Iqgap2*	IQ motif containing GTPase activating protein 2	Regulation of actin cytoskeleton	NM_027711	7.42 × 10^−5^	2.7 ↑
*Itga1*	Integrin alpha 1	Integrin signaling	NM_001033228	2.50 × 10^−2^	0.58 ↓

**TABLE 4:** Selected differentially regulated Wnt signaling genes upon vibration treatment in MC3T3-E1 cells under estrogen-free conditions.

**Gene**	**Description**	**Pathways**	**GenBank accession number**	***p*-value**	**Fold change**
*Axin2*	Axin 2	Wnt signaling pathway	NM_015732	4.13 × 10^−5^	0.24 ↓
*Dkk2*	Dickkopf WNT signaling pathway inhibitor 2	Wnt/LRP6 signaling pathway	NM_020265	1.59 × 10^−5^	0.32 ↓
*Fzd9*	Frizzled class receptor 9	Wnt signaling pathway	NM_010246	1.12 × 10^−5^	0.47 ↓
*Sostdc1*	Sclerostin domain containing 1	Wnt signaling pathway	NM_025312	2.71 × 10^−3^	0.24 ↓

**TABLE 5:** Selected differentially regulated microRNAs in hMSCs upon vibration.

**Gene**	**Description/Function**	**GenBank accession number**	***p*-value**	**Fold change**
*miRNA 548a-2*	Involved in alveolar bone loss	NR_030317	3.83 × 10^−3^	1.76 ↑
*miRNA 377*	Reduced osteosarcoma proliferation	NR_029869	3.326 × 10^−2^	1.54 ↑
*miRNA 219a-2*	Regulates osteogenic diff	NR_029837	1.088 × 10^−3^	1.52 ↑
*miRNA 518b*	Upregulated in PO	NR_030196	2.255 × 10^−2^	0.64 ↓
*miRNA 194-2*	Reduced osteosarcoma proliferation, increased apoptosis	NR_029829	2.645 × 10^−4^	0.43 ↓

## Discussion

Estrogen is a key hormone for bone homeostasis, therefore, estrogen-deficiency in postmenopausal females or after ovariectomy in rodents lead to osteoporosis development. Furthermore, fracture healing was found to be disturbed in osteoporotic women and in female mice after ovariectomy because of impaired endochondral bone formation (Beil et al., 2010; Wehrle et al., 2015; Haffner-Luntzer et al., 2017; Wildemann et al., 2021). Biomechanical stimulation by whole-body vibration was proposed as a readily applicable and non-invasive treatment approach for osteoporotic fracture healing. However, preclinical studies demonstrate that the estrogen and ERα signaling play an important role in the response to vibration treatment. Therefore, the aim of this study was to further investigate the target cells of vibration during fracture healing and the role of estrogen-dependent and -independent ERα signaling particularly in cells of the osteoblast lineage.

In the present study, mice lacking the ERα receptors on osteoblast-lineage cells were protected from ovariectomy-induced compromised fracture healing, indicating that the ERα in these cells contributes to the pathomechanisms of osteoporosis-induced impaired bone repair. As expected, osteoblast number and activity were reduced in ERα^fl/fl^ mice after ovariectomy, whereas these parameters were unaltered in OVX ERα^fl/fl; Runx2Cre^ mice. This suggests that ligand-independent ERα signaling negatively regulates osteoblast formation and activity in the fracture callus of estrogen-deficient mice during bone regeneration. Regarding the role of ERα during fracture healing, to our knowledge, there is only one other study using mice with an ERα deletion in mature osteoblasts and osteocytes that uses a monocortical defect model on the tibiae of female mice (Ikedo and Imai, 2021). In agreement with our findings, they also reported that the ERα on mature osteoblasts controls osteoblast number and osteoblast surface. However, their defect model does not represent a full fracture of the bone, thus lacking the endochondral ossification process. Another difference between the two studies is that Ikedo et al. deleted the ERα by expressing the Cre recombinase under the control of the osteocalcin promotor, whereas we used the Runx2-Cre model. In comparison to osteocalcin, which is expressed by mature osteoblasts, *Runx2* is necessary for the differentiation of osteoblastic precursors, thus the deletion of the ERα receptors on osteoblast lineage cells affected these cells earlier (Komori et al., 1998).

The presence or absence of estrogen plays a crucial role in regulating the mechanotransduction pathways upon LMHFV. In this study, we confirmed that while the application of LMHFV impaired bone formation in the fracture callus of non-OVX ERα^fl/fl^ mice, it improved the healing outcome in OVX ERα^fl/fl^ animals, consistent with previous reports (Stuermer et al., 2010; Cheung et al., 2012; Wehrle et al., 2014; Wehrle et al., 2015; Chow et al., 2016; Haffner-Luntzer et al., 2018a). We demonstrated that the vibration-induced effects on fracture healing are mediated via osteoblast lineage-specific ERα signaling because the effects of vibration on bone repair were abolished or even reversed in osteoblast lineage-specific ERα-KO animals (ERα^fl/fl; Runx2Cre)^. On a cellular level, osteoblast number and surface were unaffected by LMHFV in non-OVX KO animals, whereas a reduction of both parameters was found in the fracture callus of OVX ERα^fl/fl; Runx2Cre^ mice that were subjected to LMHFV. Histological characterization of the fractures revealed the persistence of residual cartilage in OVX ERα^fl/fl; Runx2Cre^ mice that were subjected to vibration, consistent with the impaired healing and the poor biomechanical properties of the fractured bones. We conclude from our data that ligand-dependent ERα signaling in osteoblast lineage cells might be responsible for the negative effects of LMHFV in estrogen-competent mice and that ligand-independent ERα signaling in osteoblast lineage cells is important for the positive effects of LMHFV under estrogen-deficient conditions. The reasons why deletion of the ERα specifically on cells of the osteoblast lineage even reversed the effects of vibration in OVX mice remains to be elucidated.

To further investigate which pathways might contribute to the vibration-mediated improved bone healing under estrogen-deficient conditions, we performed microarray-based gene expression analysis *in vitro* in vibrated osteogenic cells. We identified the Cox2/PGE_2_ and Wnt pathway as critical signaling pathways that are regulated by LMHFV under estrogen-deficient conditions in MC3T3-E1 cells. Specifically, our array data revealed that Cox2, involved in prostaglandin biosynthesis, and the prostaglandin receptor EP4 were significantly upregulated. Cox2 is well known to be induced by mechanical strain and was recently reported to be regulated by LMHFV *in vitro* (Haffner-Luntzer et al., 2018b). In this study, siRNA knockdown of ERα in MC3T3 cells followed by LMHFV both with and without estrogen supplementation was performed, showing that Cox2 expression is significantly increased upon LMHFV in the absence of estrogen and significantly reduced by LMHFV in the presence of estrogen (Haffner-Luntzer et al., 2018b). Furthermore, it was demonstrated by Haffner-Luntzer et al., that these effects of LMHFV on Cox2 expression were both abrogated by siRNA knockdown of ERα. Additionally, the metabolic activity was also upregulated by vibration in an estrogen-depleted environment, but not in the presence of estrogen. In primary osteoblasts isolated from C57BL/6J wildtype mice, the significant downregulation of Cox2 upon vibration in estrogen-supplemented medium was confirmed, whereas this effect was abrogated in primary osteoblasts derived from ERα general KO mice (Haffner-Luntzer et al., 2018b). Because of these data and the fact that Cox2 upregulation is indispensable for mechanotranduction, we performed all *in vitro* experiments in an estrogen-depleted environment and with the same vibration regime that was used in our previous study (Haffner-Luntzer et al., 2018b).

Studies using mechanical loading via dynamic cell stretching (Liedert et al., 2010) or via fluid shear stress (Yeh et al., 2010) have shown a synergistic effect of estrogen and mechanical stimulation regarding Cox2 upregulation in osteoblasts, however, this effect might not account for vibration and seems to be dependent on the type of stimulus. Regarding fracture healing, studies with Cox2-selective inhibitors or Cox2-KO mouse models demonstrated a significantly disturbed bone healing (Simon et al., 2002; Zhang et al., 2002), indicating an important role of this pathway during bone regeneration. This is supported by a study with local Cox2 overexpression at the fracture site revealing an acceleration of fracture healing (Lau et al., 2013). Furthermore, the application of EP4 receptor agonist was shown to rescue impaired fracture healing in Cox2-KO mice (Xie et al., 2009), which highlights the importance of Cox2-and EP4 for endochondral bone repair. These studies together with our array data might explain the improved bone healing by LMHFV in our OVX ERα^fl/fl^ animals, possibly mediated via Cox2 upregulation. We have further provided evidence for Cox2 upregulation upon LMHFV in hMSCs and that several miRNAs might also be involved in the effects of LMHFV on hMSCs. However, while these findings regarding the regulation of miRNAs upon vibration are interesting, they still require further detailed investigation in future projects.

Regarding the molecular mechanism that is involved in Cox2-induced osteogenesis, it has been recently demonstrated that the Cox2/PGE_2_/EP4 axis is involved in mechanosensing of primary cilia after LMHFV (Li et al., 2021). Primary cilia act as important mechanosensors on osteoblasts, with EP4 was found to be expressed at the bases of these cilia. Under LMHFV treatment, MC3T3-E1 cells were shown to upregulate EP4 and displayed a lower number and length of cilia. By blocking the EP4 receptor, primary cilia mechanotransduction and LMHFV-mediated osteogenesis were abrogated, suggesting a crosstalk of primary cilia and the Cox2/PGE_2_/EP4 pathway to facilitate mechanotransduction signaling.

One signaling pathway that might be activated by the Cox2/PGE_2_/EP4-mediated mechanotransduction is the Wnt signaling pathway. Wnt signaling plays a crucial role in bone homeostasis and fracture healing, whereby an activated Wnt signaling is associated with an improved fracture repair (Kim et al., 2007; Chen and Alman, 2009), whereas inhibited Wnt signaling led to delayed fracture healing (Huang et al., 2012; Beier et al., 2014; Haffner-Luntzer et al., 2014; Liedert et al., 2014; Haffner-Luntzer et al., 2016; Teng et al., 2018). In our microarray data, we found a downregulation of canonical Wnt/β-catenin pathway inhibitors (*Axin2, Sostdc1, Dkk2*), indicating that the canonical Wnt pathway is activated by LMHFV. This was further substantiated by the study of Gao et al. showing that vibration treatment of primary osteoblasts resulted in an upregulation of the Wnt signaling genes *Wnt3a, Lrp6* and *β-catenin in vitro* (Gao et al., 2017). By contrast, the Wnt receptor Fzd9, which is essential for activating the non-canonical Wnt pathway, was downregulated. It is known that Fzd9-KO mice display an impaired bone strength during fracture healing, whereas it was found that the canonical Wnt signaling was unaffected by Fzd9 deficiency in osteoblasts (Heilmann et al., 2013). Therefore, the mechanism involved in the downregulation of Fzd9 and its effects on osteogenesis after LMHFV is not completely clear. Even so, our data strongly suggests that the canonical Wnt signaling is activated upon LMHFV via downregulation of Wnt inhibitory molecules. Several studies suggested a strong association between Cox2 and the Wnt/β-catenin signaling, showing that Cox2 regulates the transcriptional and translational levels of β-catenin (Zheng et al., 2019) and that Cox2 is important for osteoblast maturation via Wnt pathway genes (Nagano et al., 2017). Other studies reported that PGE_2_ is able to induce bone formation via promoting the production of insulin-like growth factor 1 (IGF-1) and activating the protein kinase B (Akt) (Sunters et al., 2010; Xia et al., 2010). Since there is no data available regarding a possible connection between Cox2/PGE_2_ and Wnt signaling in the context of LMHFV, we did perform *in vitro* experiments with EP4 or Cox2 inhibitors and could prove that the presence of Cox2 or EP4 is crucial for LMHFV-induced *Lef1* upregulation in MC3T3-E1 cells *in vitro*, indicating an interaction of both pathways upon LMHFV.

We therefore hypothesize that mechanotransduction *in vivo* first requires estrogen-independent ERα signaling in osteoblast lineage cells, which subsequently triggers Cox2 expression and PGE_2_ production that might lead to differentially expressed Wnt-related target genes. Ultimately, this might favor an activation of the Wnt pathway, which could further promote fracture healing.

A limitation of our study was that we used the OVX model to induce estrogen-deficiency. Because the ovaries are not the only source of estrogen, small amounts of circulating estrogen might still be present in OVX mice (Haisenleder et al., 2011). Another limitation is that we used a diaphyseal femur fracture model which does not reflect the most common fracture sites (vertebral compression fractures, hip fractures, distal radius fractures, proximal humerus fractures) or the fracture type (metaphyseal), that commonly occur in postmenopausal women. However, it was shown that molecular mechanisms did not differ greatly between diaphyseal and metaphyseal fracture healing (Haffner-Luntzer et al., 2020).

Furthermore, with the performed three-point bending test, we did not evaluate ultimate bone strength. This test is also critically dependent on the callus geometry, which was not consistent between all mice. Therefore, a torsion test would be the best way to analyze mechanical stability, however, there is also evidence that torsion testing is not superior to our three-point bending test in small rodents (Steiner et al., 2015). Also, it should be also considered that hypertrophic chondrocytes in the cartilaginous fracture callus undergoing transdifferentiation to osteoblasts (Zhou et al., 2014) might express the Cre recombinase under the Runx2-promotor and might also be affected by the KO. Therefore, we cannot discriminate in our model between the effects of LMHFV on endochondral ossification and direct ossification, which both occur in the fracture callus in the chosen surgical model. Another limitation is that we did not compare all eight experimental groups to each other to investigate direct interaction between genotype and OVX/vibration treatment. This was due to considerations to reduce the number of needed animals.

In conclusion, our study suggests a critical role of the ERα in osteoblast lineage cells during mechanotransduction in the fracture callus both under estrogen-deficient and -sufficient conditions. This work provides further insights into the molecular mechanism responsible for the effects of estrogen or estrogen-deficiency on mechanostimulation during fracture healing and might result in improved treatment strategies for osteoporotic fracture patients, because we demonstrated that in addition to the estrogen status, the presence of ERα is important for mediating the positive effects of LMHFV on fracture healing. To ensure a safe treatment with LMHFV, further research is needed to define eligible patient cohorts that would benefit most from this treatment.

## Data Availability

The original contributions presented in the study are included in the article/Supplementary Material, further inquiries can be directed to the corresponding author.

## Supplementary Material

Supplementary Figure S1 : Osteoblasts, osteocytes and hypertrophic chondrocytes are affected by the deletion of ERα, but not bone marrow cells. Immunohistochemical staining for ERα were performed on paraffin sections of **(A,C)** ERα^fl/fl^ and **(B,D)** ERα^fl/fl^ ^; Runx2Cre mice^. Representative images of the fracture callus, growth plate and bone marrow are shown. Scale bar = 100 μm in overview images of the fracture callus and 50 μm in images 1–9; g= gap.

Supplementary Figure S2 : Influence of OVX treatment on trabecular bone parameters. μCT analysis of the left femora was performed to determine the relative bone volume (BV/TV) in the trabecular bone compartment of ERα^fl/fl^ **(A)** and ERα^fl/fl; Runx2Cre^ **(B)** mice. The trabecular number (Tb.N) was also determined in ERα^fl/fl^ **(C)** and ERα^fl/fl; Runx2Cre^ **(D)** mice. Data are shown as box-and-whisker plots (with median and interquartile range) from max. to min., showing all data points. *indicates significant effects with *p* ≤ 0.05; *n* = 4–5.

Supplementary Figure S3 : Influence of the respective genotype on fracture healing outcome on day 21. Biomechanical testing data (bending stiffness), bony bridging score and BV/TV of the fracture calli from control groups were directly compared between ERα^fl/fl^ and ERα^fl/fl; Runx2Cre^ mice. Data are shown as box-and-whisker plots (with median and interquartile range) from max. to min., showing all data points. *indicates significant effects with *p* ≤ 0.05.

Supplementary Figure S4 : Nondetailed STRING protein-association network (action) derived from the MC3T3-E1 microarray data in high quality.
