# Supplementary figures and images for: Estrogen Receptor α Signaling in Osteoblasts is Required for Mechanotransduction in Bone Fracture Healing
Source: Front Bioeng Biotechnol. 2021 Dec 7;9:782355. doi: 10.3389/fbioe.2021.782355 (PMC8689144; doi:10.3389/fbioe.2021.782355)

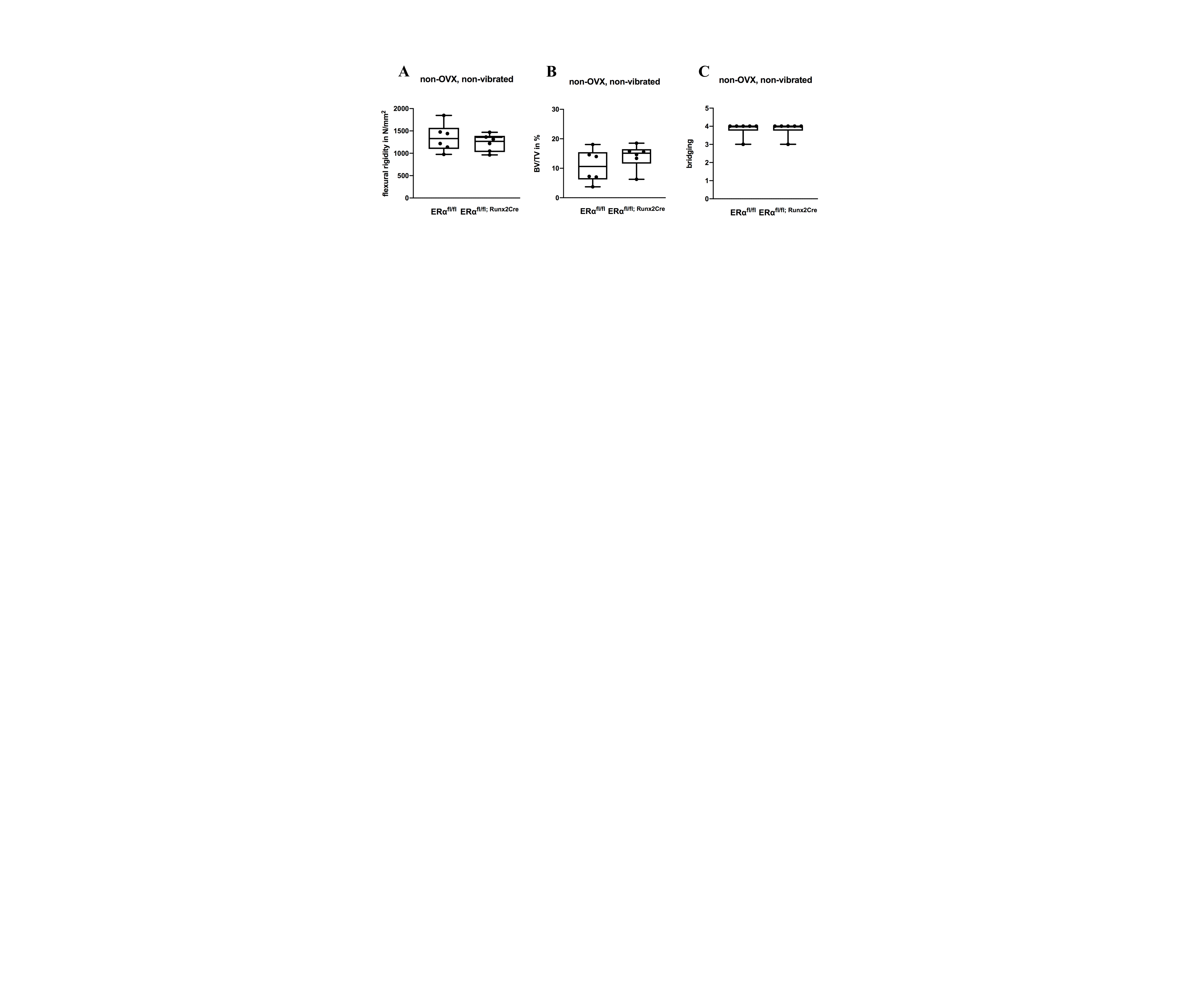

Supplement: Supplementary file 1 [file Image3.JPEG]

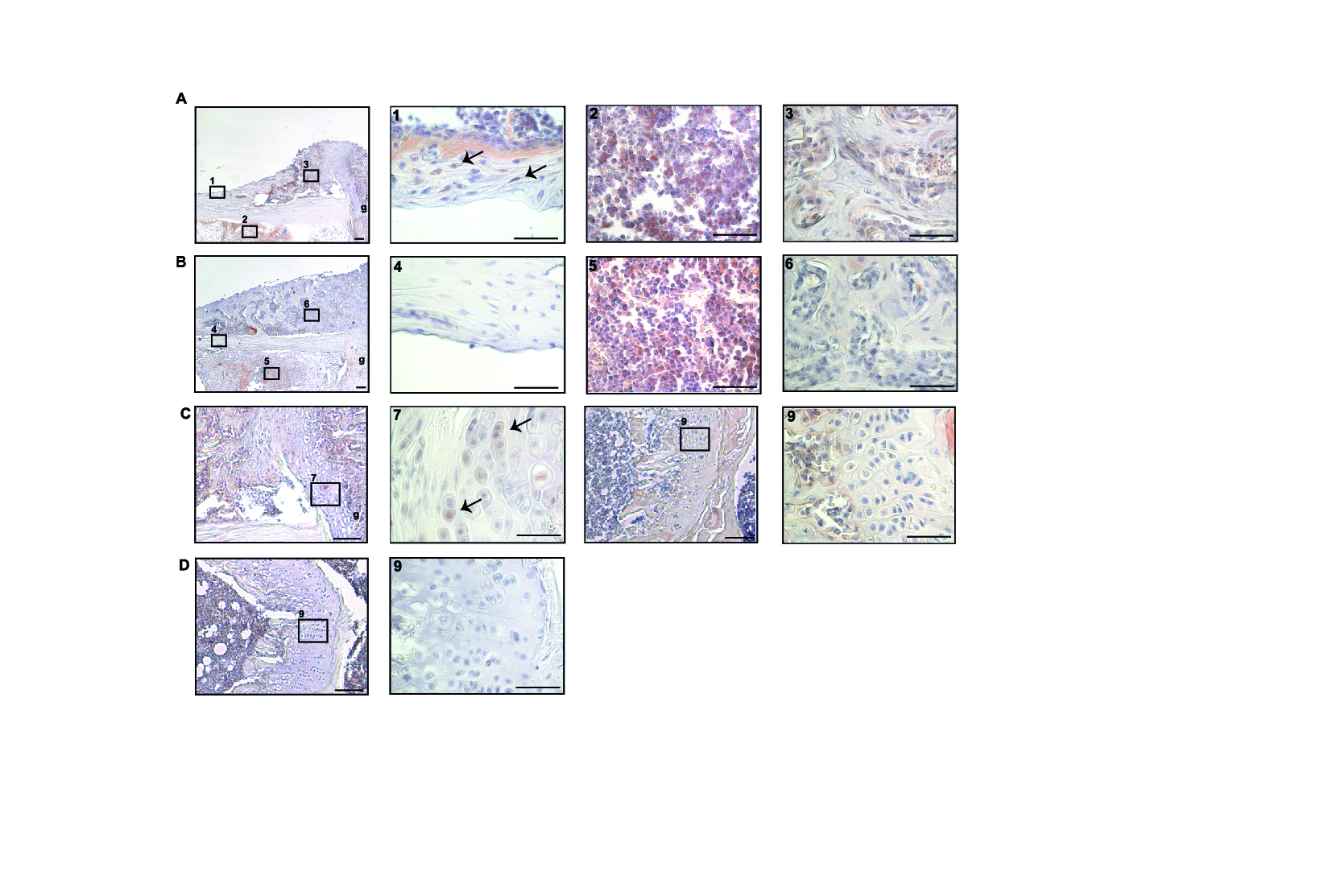

Supplement: Supplementary file 2 [file Image1.JPEG]

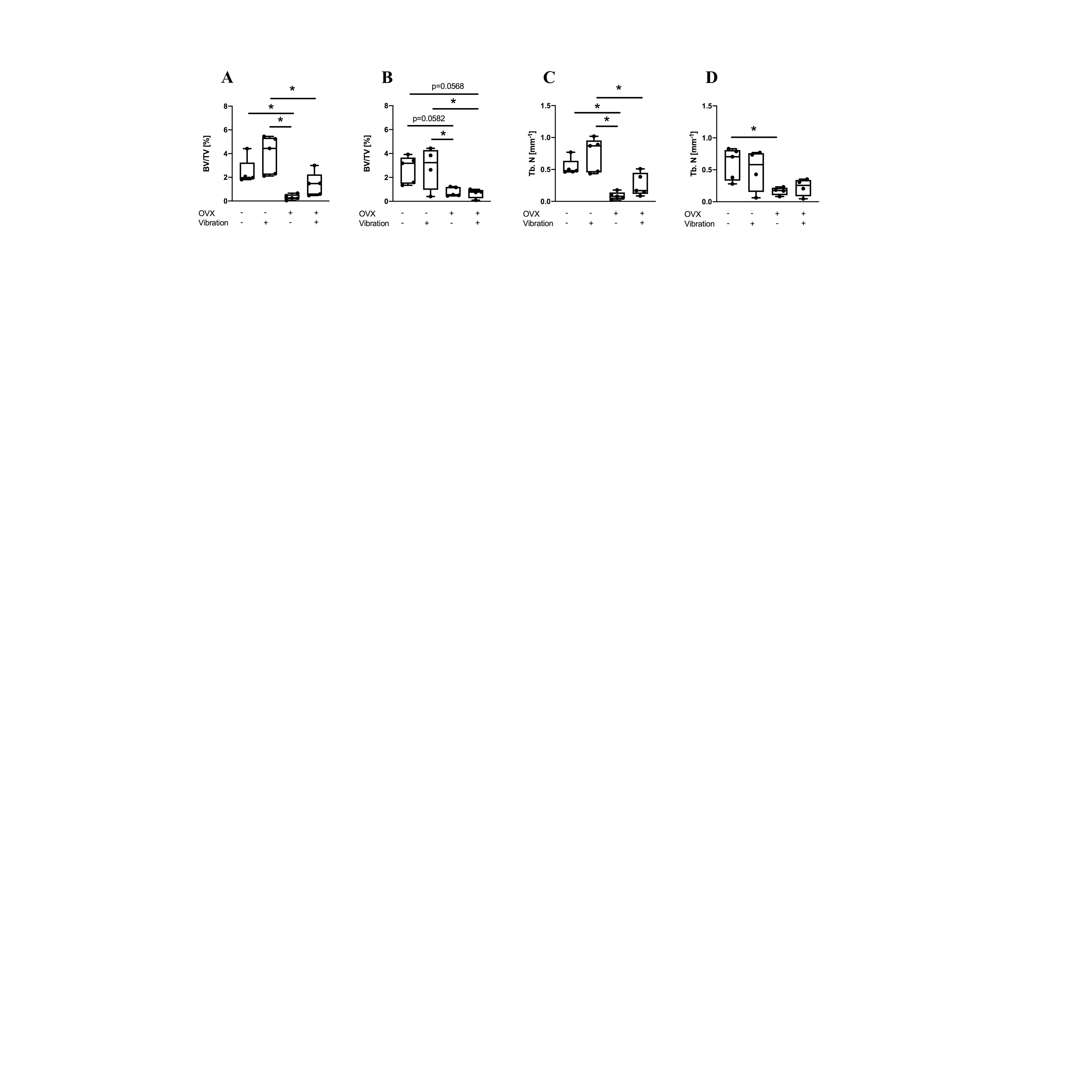

Supplement: Supplementary file 3 [file Image2.JPEG]

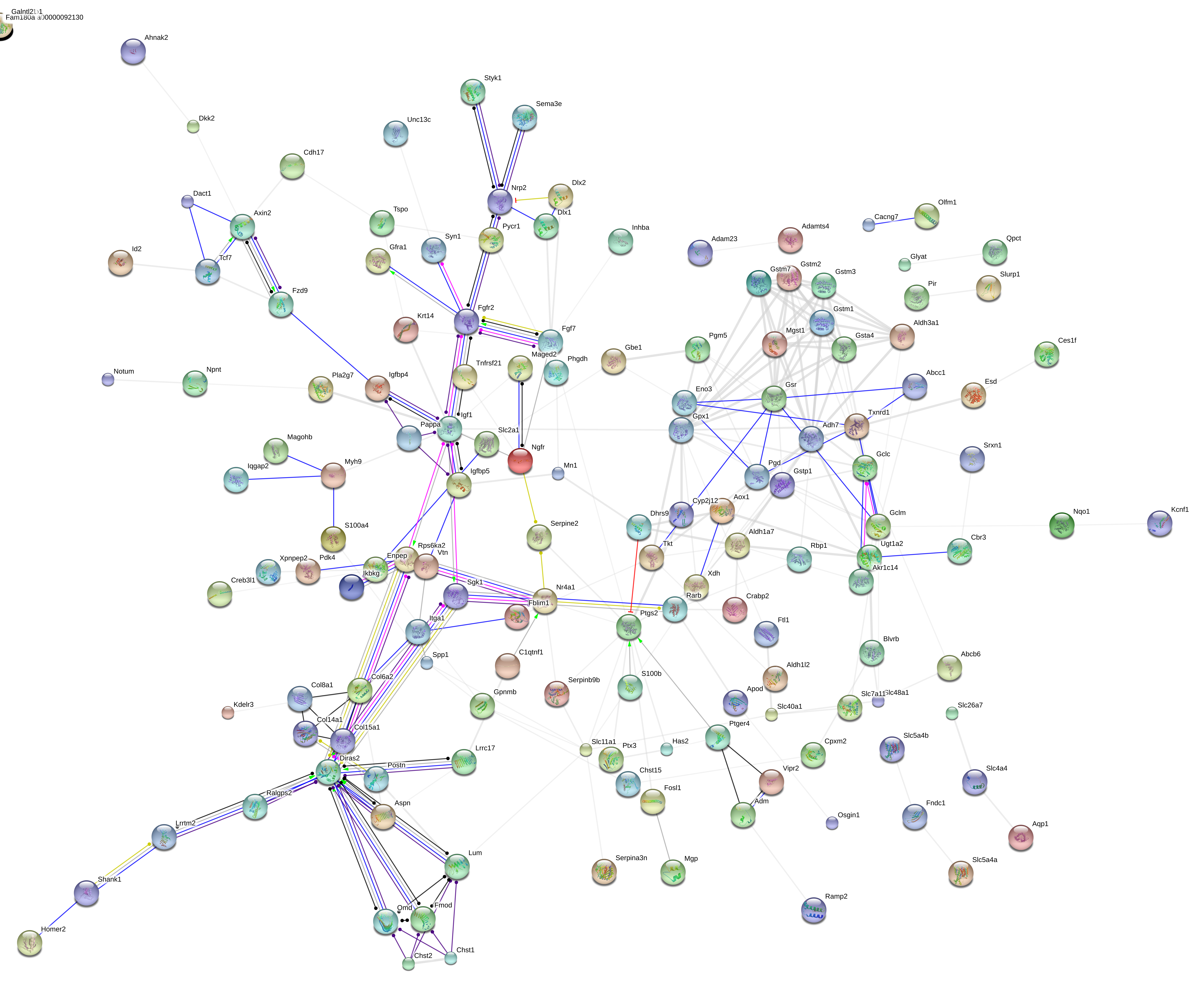

Supplement: Supplementary file 4 [file Image4.PNG]
